# Bilateral Endobronchial Metastasis in Postoperative Stage I Pulmonary Adenocarcinoma

**DOI:** 10.1155/DTE.6.141

**Published:** 2000

**Authors:** Masato Kanzaki, Takamasa Onuki, Takayuki Tatebayashi, Kunihiro Oyama, Masahide Murasugi, Sumio Nitta

**Affiliations:** Department of Surgery I Tokyo Women's Medical University 8-1 Kawada-cho, Shinjuku-ku Tokyo 162-8666 Japan

## Abstract

We reported a case of bilateral endobronchial metastasis in postoperative synchronous adenocarcinoma. Twenty months ago, a 63-year-old man underwent combined operation. Biopsy was performed, histological diagnosis of pulmonary adenocarcinoma. When surgery is not indicated because the patient has decreased pulmonary function and contralateral metastatic lesions, the Nd–YAG laser has been used to treat focal malignancy of the trachea and mainstem bronchi, and the laser has been effective, especially in patients with inoperable lesions.


*Diagnostic and Therapeutic Endoscopy*, Vol. 6, pp. 141–145
Reprints available directly from the publisher
Photocopying permitted by license only

© 2000 OPA (Overseas Publishers Association) N.V.
Published by license under
the Harwood Academic Publishers imprint,
part of The Gordon and Breach Publishing Group.
Printed in Malaysia.

*A Case Report*

### Bilateral Endobronchial Metastasis in Postoperative Stage I Pulmonary Adenocarcinoma

MASATO KANZAKI*, TAKAMASA ONUKI, TAKAYUKI TATEBAYASHI, KUNIHIRO OYAMA, MASAHIDE MURASUGI and SUMIO NITTA

*Department of Surgery I, Tokyo Women's Medical University, 8-1 Kawada-cho, Shinjuku-ku, Tokyo 162-8666, Japan*

*(Received 24 May 1999; Revised 25 November 1999; In final form 27 December 1999)*

**We reported a case of bilateral endobronchial metastasis in postoperative synchronous adenocarcinoma. Twenty months ago, a 63-year-old man underwent combined operation. Biopsy was performed, histological diagnosis of pulmonary adenocarcinoma. When surgery is not indicated because the patient has decreased pulmonary function and contralateral metastatic lesions, the Nd–YAG laser has been used to treat focal malignancy of the trachea and mainstem bronchi, and the laser has been effective, especially in patients with inoperable lesions.**

*Keywords:* Endobronchial metastasis, Neodymium–yttrium aluminum-garne laser (Nd–YAG laser), Pulmonary adenocarcinoma, Synchronous adenocarcinoma

Bilateral endobronchial metastasis is rare. Postoperative endobronchial metastasis is occasionally reported, even when the bronchial stump is not invaded by lung cancer and the lesions are completely excised pathologically. Surgical treatment was not indicated in our patient, who had multiple metastases. Laser procedures were used to open obstructed airways.

### CASE REPORT

A 63-year-old man was admitted to the hospital. Twenty months before he had been referred to us because an abnormal shadow had been observed in the right lower lung field during a preoperative examination for adenocarcinoma of the stomach, lung cancer was diagnosed because adenocarcinoma cells were detected by brushing cytology with a bronchofiberscope. Both of the patient's cancers were early stage tubular adenocarcinomas. The gastric cancer was localized in the submucosa. The pulmonary adenocarcinoma was not associated with enlarged mediastinal lymph nodes on computed tomography of the chest. We made a diagnosis of double primary cancer based on the patient's clinical course and pathological findings.

* Corresponding author. Tel.: +81 3 3353 8111, ext. 31125. Fax: +81 3 5269 7333.

Combined right lower lobectomy and subtotal gastrectomy followed by lymph node dissection were performed in October 1995. The histopathological diagnosis was stage IB (T2 N0 M0) adenocarcinoma of the lung and was stage I adenocarcinoma of the stomach. The lesions were completely resected.

The patient subsequently attended the outpatient department of our hospital about once a month for a regular checkup.

A chest X-ray taken in the outpatient department in June 1997 was unremarkable, with no deviation of the trachea or bronchi. Computed tomography of the chest, however, revealed two 1.0-cm tumors, in the right middle bronchus and the other in the left main bronchus. There were no enlarged mediastinal lymph nodes. No abnormal findings were observed around the trachea or bronchi (Fig. 1). Bronchoscopic examination revealed bronchial tumors that almost totally occluded the right middle lobe

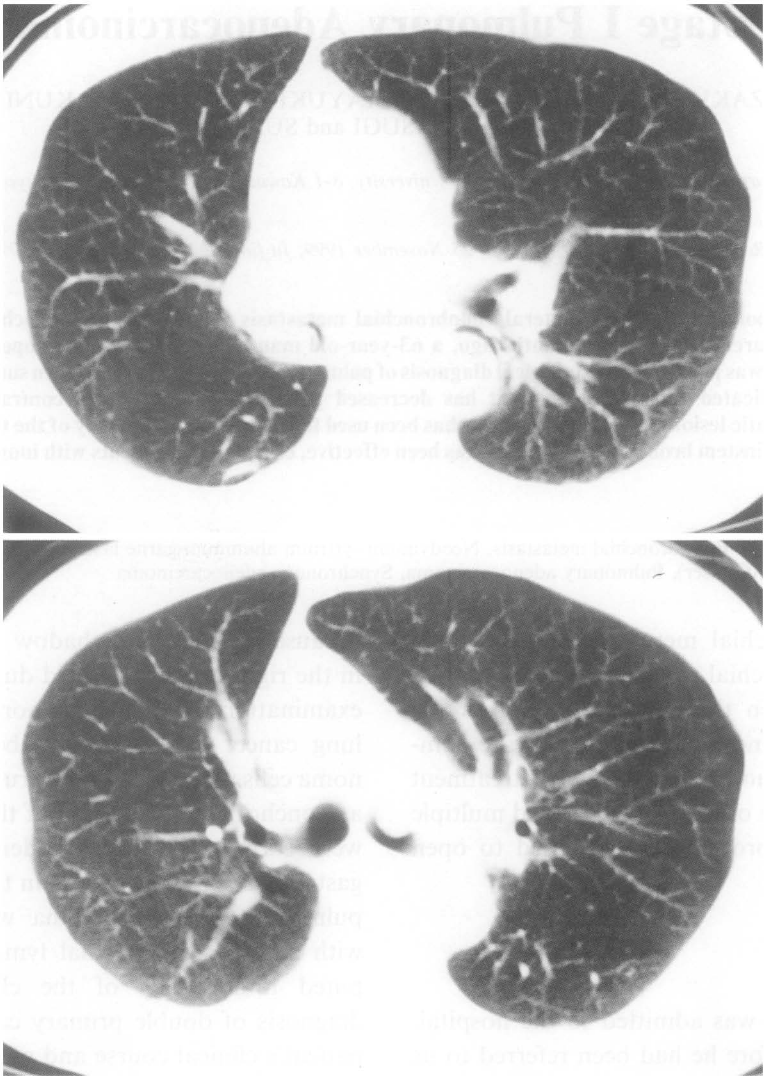

**FIGURE 1** Chest computed tomography revealed two 1.0-cm tumors, in the right middle bronchus and left main bronchus.

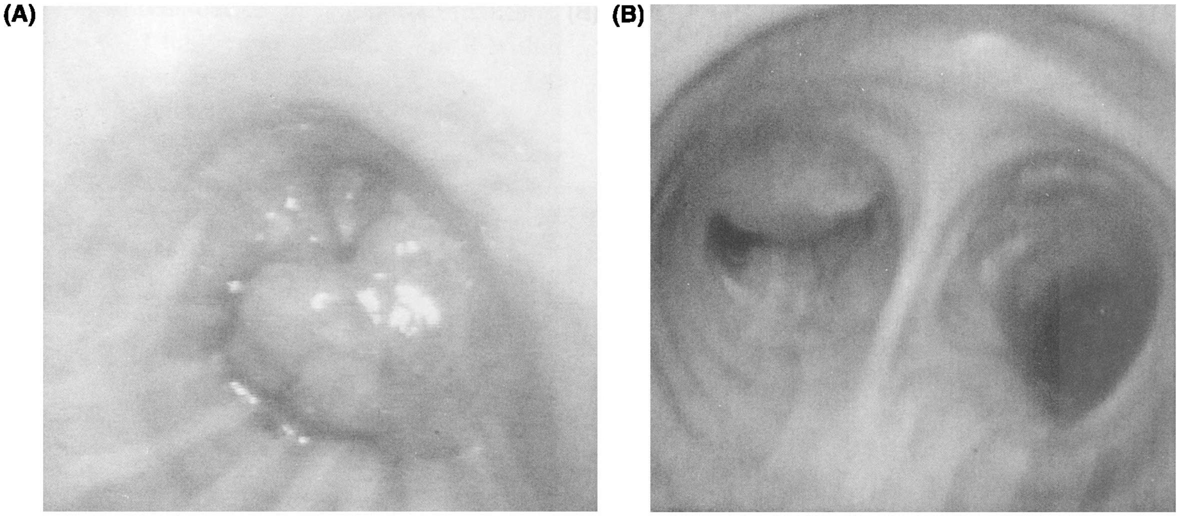

FIGURE 2 Bronchoscopic finding revealed the bronchial tumors with total occlusion of the right middle lobe bronchus (A) and two-thirds obstruction of the left main bronchus (B).

bronchus and two-thirds occluded the left main bronchus. The lesions were in the form of polyps (Fig. 2).

Although the patient was asymptomatic and the physical examination on admission was unremarkable, he had undergone lobectomy and multiple metastases had been recognized. In view of his post-lobectomy status and poor pulmonary function, we chose to cauterize the tumors with an Nd–YAG laser and to them by electrosurgical snaring through a bronchofiberscope to maintain his present pulmonary function. Since the obstructed airways were to be opened, bronchofiberscopy was performed under general anesthesia. First, the tumor that had in the right middle lobe bronchus was resected for biopsy purposes. Microscopic examination of the specimen showed moderately differentiated adenocarcinoma. Tubular and papillary growth was observed, and the pathohistological findings were similar to those of pulmonary adenocarcinoma.

The endobronchial metastatic lesions were safely and completely cauterized with the Nd–YAG laser through the bronchoscope, and part of the left main bronchus cancer was resected by electrosurgical snaring (Fig. 3).

The final histopathologic diagnosis was bronchial metastasis of pulmonary adenocarcinoma (Fig. 4). The postoperative course was uneventful, and the patient was discharged ten days after the laser therapy. He is currently alive and well with no evidence of recurrent disease as of the 18-month follow-up examination.

### CONCLUSION

Lung cancer almost always metastasizes to liver, lungs, and bone. However we encountered a case of stage IB postoperative adenocarcinoma of the lung with rare post-lobectomy bilateral endobronchial metastasis in the 20th month after right lower lobectomy before clinical signs or symptoms were manifested [1,2]. Complete resection of the lung cancer was achieved at the first operation because no residual cancer cells in the endobronchial stump or lymph node metastasis was detected.

The gastric cancer was stage I, and it was also completely resected. Biopsy was performed, and the pathohistological examination of tumors revealed adenocarcinoma. Both cancers had the same histopathological features. It was difficult to determine whether the metastases originated in the lung cancer

(A) (B)

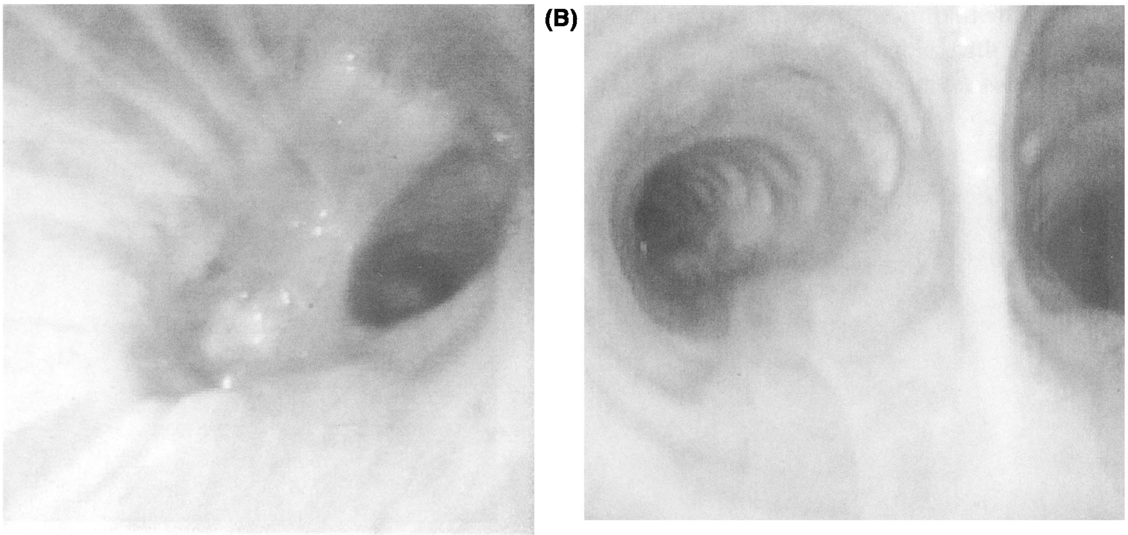

FIGURE 3 Postoperative bronchoscopic findings showed the obstructed airways to be opened (A, B). A granuation was recognized at the bronchial stump (A).

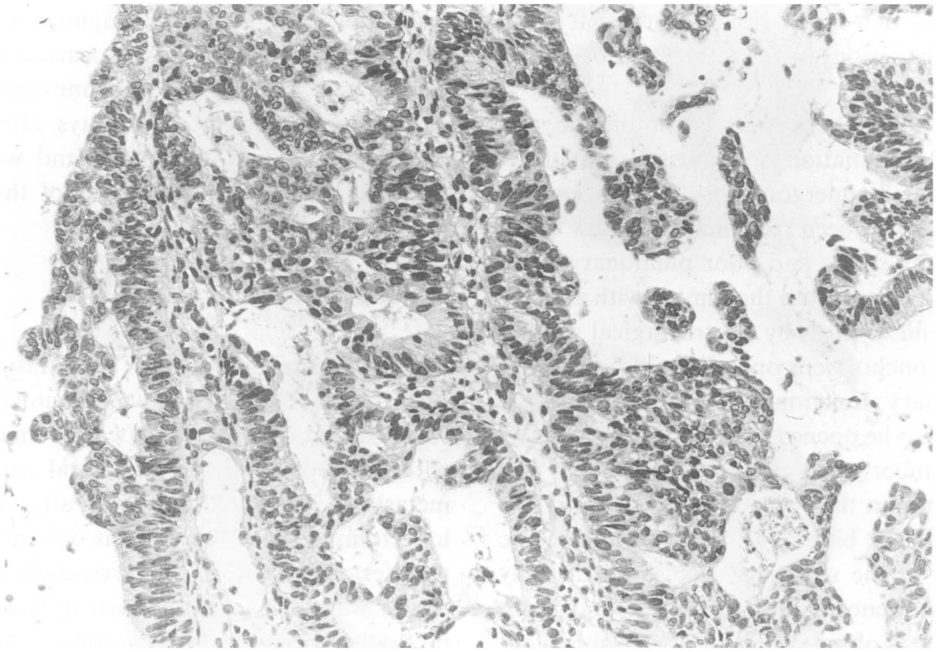

FIGURE 4 Microscopic appearance (H & E ×40) demonstrated moderately differentiated adenocarcinoma.

or the gastric cancer. However, the results of repeated studies showed that the cell subtype of the lung cancer was papillary growth as well as tubular growth, and when the clinical findings were added, the gastric cancer was early cancer and confined to the submucosal layer, and the frequency and likelihood of metastasis are smaller than in lung cancer.

Martini and associates have reported that most recurrences of lung cancer are discovered within the two years after surgical removal [3], and the pathological studies in our patient revealed that the endobronchial metastase were from the lung cancer.

When surgery is not indicated because the patient has decreased pulmonary function and contralateral metastatic lesions, the Nd–YAG laser has been used to treat focal malignancy of the trachea and mainstem bronchi, and the laser has been effective, especially in patients with inoperable lesions [4]. We performed Nd–YAG laser therapy on our patient to decrease the volume of the cancers and it was useful in opening the obstructed airways.

Computed tomography of the chest did not show any enlarged mediastinal lymph nodes or abnormal findings around the trachea or bronchi at the time the endobronchial metastase were discovered.

These findings rule out growth of the cancer along the bronchial mucosa, direct invasion from lymph nodes, and retrograde metastasis. Heitmiller reported that the site of endobronchial metastase is the bronchial submucosa and that if the metastatic tumor is small, the overlying bronchial mucosa may be intact [5].

Schoenbaum and Viamonte reported that metastatic spread of malignant tumors to bronchi occurs by direct extension from a mediastinal lesion, invasion of a bronchus by a parenchymal mass, or direct metastasis to the wall of the bronchi, and that reaching the bronchial wall via bronchial arteries is the most important route [6]. In our patient endobronchial metastasis occurred in spite of the absence of lymph node metastasis, and the metastatic pathway was suspected of being the bronchial arteries in the bronchial wall.

When patients operated on for lung cancer have multiple endotracheal and endobronchial metastatic lesions, combined therapy consisting of Nd–YAG laser therapy and electrosurgical snaring is very useful for maintaining pulmonary function.

We believe that strict follow-up for endobronchial metastasis is needed even when the cancer is early.

### *References*

[1] Braman, S.S. and Whitcomb, M.E. Endobronchial metastasis. *Arch. Inter. Med.* 1975; **135**: 543–547.

[2] William, A.B. and James, B.D.M. Metastatic malignancies from distant sites to the tracheobronchial tree. *J. Thorac. Cardiovasc. Surg.* 1980; **79**: 499–503.

[3] Martini, N., Ghosn, P. and Melamed, M.R. Local recurrence and new primary carcinoma after resection. In Delarue, N.C. and Eschapasse, H. (Eds.) *Lung Cancer*. Philadelphia: WB Saunders, 1985; 164–169.

[4] Sakai, T., Ikeda, T., Kikuchi, K. *et al.* A case of trachial metastasis of pulmonary cancer. *J. Japanese Association for Thorac. Surg.* 1986; **34**: 1178–1181.

[5] Heitmiller, R.F., Marasco, W.J., Hruban, R.H. *et al.* Endobronchial metastasis. *J. Thorac. Cardiovasc. Surg.* 1993; **106**: 537–542.

[6] Schoenbaum, S. and Viamonte, M. Subepithelial endobronchial metastasis. *Radiology* 1971; **101**: 63–69.

